# Editorial: Community series in innate immunity: platelets and their interaction with other cellular elements in host defense and disease pathogenesis, volume II

**DOI:** 10.3389/fimmu.2026.1776088

**Published:** 2026-02-06

**Authors:** Meganathan Kannan, Firdos Ahmad, Esaki M. Shankar

**Affiliations:** 1 Blood and Vascular Biology Research Lab, Department of Biotechnology, Central University of Tamil Nadu, Thiruvarur, India; 2 Department of Basic Medical Sciences, College of Medicine, University of Sharjah, Sharjah, United Arab Emirates; 3 Infection and Inflammation, Department of Biotechnology, Central University of Tamil Nadu, Thiruvarur, India

**Keywords:** cell-cell interactions, inflammatory responses, innate immunity, microparticles, platelets

Platelets are no longer regarded as cell fragments due to their involvement in various biological functions, including their role in immune functions. Both the surface receptors and cellular components of platelets play key roles in thrombus formation, tissue repair and immunological responses (1). Beyond hemostasis, platelets serve as active mediators of inflammation and contribute to innate and adaptive immune responses (2). The platelets’ surface receptors such as Toll-like receptors (TLRs), integrin receptors, cytokine receptors play a major role during pathological manifestations (3–5). The TLRs of platelets serve as immune sensors and detect pathogens leading to immune/inflammatory responses through intracellular signaling. The integrin receptors such as CD40L and αIIbβ3 are known to necessitate cell adhesion and aggregation leading to integrin-dependent inflammation (6). Activated platelets also express cytokine receptors such as Interleukin-1 receptors (IL-1R), which links thrombosis with innate immunity. The platelets’ surface receptors also facilitate the cross-talk between platelets and circulating immune cells including monocytes and neutrophils (7). Such interactions are crucial during infection as well as coagulation. For example, the participation of platelets in neutrophil extracellular traps (NET) promotes thrombo-inflammation, leading to NET-induced immunothrombosis. Therefore, understanding the NET mechanism may aid in targeting NET-induced immunothrombosis for the development of anti-thrombotic therapy (8, 9). Further, activated platelets release cellular contents including microparticles that are key, not only to arrest the bleeding but also to accomplish innate immune responses. The platelet microparticles represent important communicators rendering immune and inflammatory responses (10). The above findings indicate that platelets have pivotal role in various pathological conditions bridging thrombosis and the immune system. The current Community Series in Innate Immunity is focused on how platelets contribute to host immunity, and has addressed key questions pertinent to platelet interactions with other cellular elements in host defense and disease pathogenesis. In order to deepen the understanding of platelets’ roles in health and immunity, we invited research related to key areas such as contribution of platelets to defense mechanisms, role of activated platelets in infection, platelet microparticles and other components as immune mediators, role of platelets during inflammation and allergies, and other relevant topics. In response to the above topics, various articles including review and original research studies have been submitted.

This Research Topic received original research from Wang et al. that studied the alterations in platelet subpopulations in liver cancer patients undergoing combined immunotherapy and targeted therapy. They studied platelet surface antigens of liver cancer patients, before and after receiving combined therapy using mass cytometry by employing CyTOF technology and observed the downregulation of CD107a+ and CD62P+ platelet subpopulations in patients, indicating weakened platelet functions in liver cancer. Further, they studied β1 integrin receptor that is crucial for platelet functions and onset of angiogenesis. The authors employed CD29 to capture the β1 integrin receptor on the surface of platelets. They demonstrated that CD29+ platelets were elevated in progressive disease group indicative of their potential to serve as predictive biomarkers to understand the efficacy of combined therapy. Furthermore, the study demonstrated that CD29+ platelets decreased with tumor remission and increased with tumor progression, suggesting their likely role as therapeutic targets.

A study from University of Nebraska Medical Center in Omaha investigated whether platelet lineage-specific deletion of integrin-β3 reduces NEC-like injury in murine neonates. The necrotizing enterocolitis (NEC) is a severe intestinal condition that primarily afflicts premature or low-birth weight neonates leading to inflamed intestine and necrosis resulting in life-threatening sepsis and peritonitis (11). NEC reportedly owns a high mortality rate and thus addressing this condition is of paramount importance. Balamurugan et al., from University of Nebraska Medical Center measured plasma levels of intestinal injury markers, inflammatory cytokines and monocyte-platelet aggregates in C57BL/6 and integrin-β3−/− mouse pups. They employed immunofluorescence, flow cytometry, multiplex assay and qRT-PCR to achieve their proposed objectives. Their study revealed that monocyte-platelet aggregation is an important pathophysiological event in NEC. The deletion of integrin-β3 resulted in inhibition of platelet-monocyte aggregation in circulating blood resulted in reduced intestinal injury. The finding adds significant value to therapeutic development against NEC.

The above study employed platelet-monocyte aggregates as an important parameter in NEC. Nonetheless, platelets are highly sensitive, and any artifact during sample preparation may likely affect the diagnosis. Trivigno et al. has taken an important step to address the methodological variables that affect the detection of platelet-leukocyte aggregates. The platelet-leukocyte aggregate is a sensitive marker affected by various factors such as type of anticoagulants, blood draw techniques, processing methods and assay temperature. They evaluated the influence of these artifacts in the outcome of platelet-leukocyte aggregates by utilizing murine blood samples. Their study observed the impact of these conditions on platelets and provided recommendations for optimizing the *in vitro* analysis of murine platelet-leukocyte aggregates. The key recommendations include the choice of anticoagulant based on the experimental conditions, standardized sample collection and processing, consistent temperature conditions and titrated agonist concentrations. Following the above recommendations in the measurement of platelet-leukocyte aggregates, this sensitive maker can be used as a marker in various clinical conditions.

Platelets have emerged as key players in the diagnosis and management of many infectious diseases. One such disease where platelets have shown to be important is tuberculosis (TB). While active TB requires care and management, the healthy-household contacts (HHCs) of active TB are at a greater risk of transmitting TB. Thus, these individuals need to be monitored to interrupt TB transmission. Selvavinayagam et al. are the first to study the biochemical and hematological profiles of HHCs of active TB. They conducted a cross-sectional study to explore the possibility of employing platelet parameters as surrogate markers of subclinical inflammation in latent tuberculosis infection (LTBI). Their study identified ESR and P-LCR as prominent surrogate diagnostic biomarkers of subclinical inflammation associated with LTBI, and when combining these two markers, the efficacy of diagnosing LTBI was increased with an AUC of 0.906. This study suggests the utility of simple, routinely available hematological indices as cost-effective diagnostic tools for identifying HHCs at higher risk of LTBI in resource-limited settings.

A review article aimed to explore the role of the immune system in chronic obstructive pulmonary disease (COPD) has been submitted by Li et al. COPD is a progressive lung condition that affects the lower airways, characterized by persistent respiratory symptoms and chronic inflammation. The study explored the role of immune dysregulation in COPD pathogenesis and discussed in depth on how macrophages, neutrophils, and T-lymphocytes contribute to COPD pathogenesis. The review also described salient features of altered cytokine signaling pathways and defective resolution of inflammation. The authors have also proposed the potential therapeutic targets of specific cytokines for immunomodulatory strategies in COPD.

Altogether, the studies submitted to the community series in innate immunity are important as they strongly suggest the involvement of platelets in host defense and disease pathogenesis. Beyond their traditional role in hemostasis and thrombosis, the platelets actively participate in immunological systems and thus, the time has arrived to consider platelets as an important component in the diagnosis and management of immune-mediated disorders.
